# The role of the Kinesin-13 family protein TbKif13-2 in flagellar length control of *Trypanosoma brucei*

**DOI:** 10.1016/j.molbiopara.2010.08.001

**Published:** 2010-12

**Authors:** Kuan Yoow Chan, Klaus Ersfeld

**Affiliations:** a Department of Biological Sciences, University of Hull, Hull HU6 7RX, UK; b Hull York Medical School, University of Hull, Hull HU6 7RX, UK

**Keywords:** Flagellum, Kinesin, Sleeping sickness, Microtubule, Axoneme

## Abstract

TbKif13-2, a member of the microtubule-depolymerising Kinesin-13 family was localised at the tip of the flagellum in *Trypanosoma brucei*. Its predicted activity suggested a role in the regulation of axonemal length. However, using gene deletion and overexpression of TbKif13-2 we show that, in procyclic *T. brucei*, this kinesin has only a very limited effect on flagellar length. Gene deletion resulted in no significant elongation of the flagellum and overexpression only slightly decreased flagellar length and the rate of growth of a new flagellum during cell division. This is in contrast to studies in *Leishmania major*, where overexpression of the TbKif13-2 homologue resulted in a significant length reduction of the flagellum. Knockout of TbKif13-2 has, however, an effect on the initial growth of the emerging new flagellum. In conclusion, we show that TbKif13-2 has only a marginal impact on flagellar length in *T. brucei*.

## Graphical abstract

**Graphical abstract:**

*Trypanosoma brucei* contains at least five microtubule-depolymerising kinesins, but the flagellar kinesin TbKif13-2 has only a moderate effect on flagellar dynamics.

**Figure:**
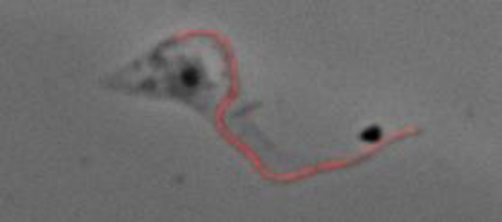

**Research highlights:**

▶ The depolymerising kinesin TbKif13-2 is localised at the tip of the mature and new flagellum of *T. brucei*. ▶ A gene deletion mutant of this kinesin has no measurable effect on the length of the flagellum. ▶ Overexpression of a myc-tagged TbKif13-2 has marginal, but significant effects on the initial rate of outgrowth of the new flagellum and on the length of the mature flagellum.

*Trypanosoma brucei* has a single flagellum which emerges from the basal body at the base of the flagellar pocket and runs along and beyond the entire cell body. In addition to the canonical 9 + 2 microtubular axoneme, the trypanosomal flagellum is characterised by the presence of the paraflagellar rod, a highly insoluble protein structure which runs parallel to the axoneme, from the flagellar pocket exit point to the distal tip of the flagellum [1]. The flagellum is highly motile and has essential roles in cellular motility, division, infectivity and possibly environmental sensing within the mammalian host and the insect vector [2]. Due to its experimental accessibility *T. brucei* has emerged as a model to study flagellar biology [3]. Flagellar assembly is dependent on kinesin and dynein motor proteins and components necessary to build a flagellum are deposited at the distal end of the structure via intraflagellar transport [4].

The completion of the trypanosome genome project has resulted in the identification of 41 putative kinesin sequences in *T. brucei* [5]. Phylogenetic analysis of the kinesin sequences in kinetoplastids indicates that there is a surprisingly large number of Kinesin-13 family members. Members of the Kinesin-13 family are non-processive motor proteins involved in the depolymerisation of microtubules at both their plus and minus ends. The trypanosome genome contains at least five Kinesin-13 members, which is more than in any other surveyed eukaryotic genomes thus far (e.g. three Kinesin-13 family members in humans). In humans, all three Kinesin-13s have mitotic functions by regulating spindle length [6] but the few protozoan Kinesin-13s characterised so far have mitotic and non-mitotic functions. Amongst these are Kinesin-13s that have a role in flagellar length regulation. This was first observed in *Leishmania major* where LmjKIN13-2 was localised to the flagellum and overexpression resulted in a shortening of the flagellum [7]. In *Chlamydomonas reinhardtii* a Kinesin-13 is involved in flagellar assembly and disassembly [8]. In *Giardia intestinalis*, the single Kinesin-13 protein is involved in regulating flagellar length, the dynamics of mitotic spindle and the cellular microtubule network during interphase [9].

Here we report an analysis of the trypanosome Kinesin-13 family member, TbKif13-2 (TriTrypDB acc no: Tb11.02.2260), the homologue to the *L. major* kinesin LmjKIN13-2. In a previous study it was shown that overexpression of a GFP-tagged LmjKIN13-2 in *L. major* and RNAi-depletion of TbKif13-2 in procyclic *T. brucei* had significant effects on flagellar length [7]. Performing RNAi on TbKif13-2 in procyclic *T. brucei*, we, however, did not observe a statistically significant effect on flagellar length. Given the limitations of RNAi (insufficient repression, off-target effects, RNAi resistance) we decided to generate a gene deletion cell line to study the function of this kinesin in more detail.

For subcellular localisation, we raised rabbit polyclonal antibodies against the native TbKif13-2 protein. However, we were unable to detect the presence of endogenous TbKif13-2 in both procyclic and bloodstream cell lines by Western blotting and immunofluorescence [10]. This was similar to the data obtained for *L. major*, where an overexpressed GFP-fusion protein was employed to localise the protein [7].

The antiTbKif13-2 antibody was able to detect the presence of an ectopically expressed, Tet-inducible myc-tagged TbKif13-2 construct at the tip of the flagellum [10]. Therefore, the failure of antiTbKif13-2 to detect the endogenous protein is due to either the absence of TbKif13-2 expression or to protein levels of TbKif13-2 below the detection threshold. The tagged kinesin, TbKif13-2myc, was clearly and exclusively detectable at the distal tip of the flagellum in interphase cells and was also observed at the tip of both the mature and developing flagellum during the cell cycle (Fig. 1A). This staining was similar to the pattern described for its orthologue LmjKIN13-2 in *L. major*, although additional staining was observed near the base of the flagellum and occasionally along the entire flagellum [7]. We did not observe the localisation of the myc-tagged TbKif13-2 at the base of the flagellum in *T. brucei*.

In *L. major* it was reported that the overexpression of LmjKIN13-2 resulted in the shortening of the flagellum in promastigotes and the depletion of TbKif13-2 in *T. brucei* procyclic cells using RNAi resulted in considerable flagellum lengthening [7]. Given our own inconclusive RNAi data (not shown) and to address the question whether TbKif13-2 is involved in the regulation of the flagellar length, a procyclic cell line where both copies of TbKif13-2 genes were deleted was generated. The successful deletion of both TbKif13-2 alleles was confirmed using Southern blot (Figure S1A) and RT-PCR from RNA isolated from knockout cell lines (Fig. 1B). The growth rates of control cells, myc-overexpressors and gene knockout cells were indistinguishable. The flagellar length of control PC449, TbKif13-2myc induced overexpressors and TbKif13-2 knockout cells was analysed by staining with the anti-PFR monoclonal antibody L8C4 and the length of the immunofluorescence signal was measured. Overlays of phase contrast and PFR immunofluorescence signal showed that in all cells the PFR extended to the distal tip of the flagellum and could therefore be used as a reliable measurement of total flagellar length (not shown). Flagellar length comparisons (Fig. 1D) indicated that there was no significant flagellar length increase (*p* = 0.065) between TbKif13-2 knockout cells (19.8 ± 2.3 μm) when compared to control cells (19.4 ± 2.2 μm). There was, however, a small but significant decrease in flagellar length in TbKif13-2myc induced, overexpressing cells (18.5 ± 3.8 μm) when compared to control cells (*p* = 0.002). This is, to some extent, compatible to the observation of the reduction of flagellar length in *L. major* promastigotes expressing a LmjKIN13-2-GFP construct, although the effect was much more pronounced in *L. major* than in *T. brucei* (52–70% decrease in *L. major* versus 5% in *T. brucei*). Possible explanations for this difference could be fundamental differences in flagellar length regulation in the two kinetoplastid species or different levels of overexpressed proteins in both organisms. In control cells however, TbKif13-2 was not found to play a significant role in flagellum length regulation as there was no significant difference in flagellar length of control and TbKif13-2 knockout cell lines.

To examine if TbKif13-2 was involved in flagellar assembly and growth rather than maintaining a balance-point equilibrium [11] after the maximum length had been reached, we examined the dynamics of the outgrowth of the new flagellum that is assembled during the cell cycle. To correlate flagellar growth with cell cycle progression during assembly of a new flagellum the distance between the dividing kinetoplasts was used as a parameter. The kinetoplast distance was chosen as it was easily visualised using DAPI. Also, kinetoplast segregation is functionally and spatially linked to basal body segregation [12,13]. Measurements of the length of the new flagellum and the distance between the two kinetoplasts were taken from control P449, TbKif13-2myc induced and TbKif13-2 knockout cells (Figs. 1E and S1B). A statistical analysis (ANCOVA test) indicated that there was a significant difference (*p* < 0.001) in the lines of best fits and a post hoc test with Sidak correction [14] indicated that both TbKif13-2myc induced and TbKif13-2 knockout cells had significantly different (*p* = 0.017 and 0.001, respectively) lines of best fit when compared to control cells. Between control and TbKif13-2myc overexpressors a different slope and between control and knockout a parallel shift was observed. Student's *t*-tests comparing the slope of the line of best fit of control against TbKif13-2myc overexpressing cells indicate that there was a significant difference (*p* = 0.005) but there was no significant difference between the slopes of control and TbKif13-2 knockout cells (*p* = 0.938). In relation to flagellar growth this indicates that the initial seed of the new flagellum is established earlier or more rapidly in cells lacking TbKif13-2, but that the subsequent growth rate is independent of its presence and does not differ between control and TbKif13-2 knockout cells. As for the effect of overexpression, the difference in length is established continuously during flagellar growth.

Extrapolation of the lines of best fit for control and overexpressors would result in a difference of at least 30% between the longer control and the shorter TbKif13-2myc overexpressing cells. This was, as explained above, not the case as the final flagellar length of the overexpressor is only 5% shorter. This theoretical discrepancy can be explained by non-linear growth rates during flagellar outgrowth. Such non-linear growth rates have been observed in classical studies of *Chlamydomonas* flagellar dynamics, but also in *T. brucei* [12,15].

Our data show that TbKif13-2 is non-essential for the survival of procyclic trypanosomes *in vitro* and the absence of TbKif13-2 does not result in flagellum lengthening or effect the rate of flagellum growth in dividing trypanosomes. Also, the staining of the axoneme using antibody Mab25 [16] in TbKif13-2 knockout cells was indistinguishable between knockout and control and there were no observable gross changes in trypanosome motility (results not shown). Our work involving the expression of TbKif13-2myc in procyclic cells represents an extension of previously published data for *L. major* and *T. brucei* [7]. However, in contrast to previously published results employing RNAi to partially deplete TbKif13-2 in *T. brucei*, gene knockout cells did not have a similar flagellar phenotype. The reason for this discrepancy is unknown but may be attributed to the different approach used in depletion of TbKif13-2 expression (gene knockout in contrast to RNAi) in procyclic cells. Also, it is of course important that localisation in *L. major* and *T. brucei* in this and the study by Blaineau et al. [7] was only achieved after overexpressing epitope-tagged, ectopic constructs. It is therefore unknown whether this kinesin is expressed in the life cycle stages examined (promastigote *L. major* and procyclic and bloodstream *T. brucei*). In particular, it would be interesting to study its role in epimastigote stages of *T. brucei*, where an extensive remodelling of the flagellum takes place as a prerequisite to a differentiation process [17]. However, the subtle, but significant, effects of kinesin gene deletion on flagellar outgrowth dynamics argues that this kinesin has a functional role at least in the procyclic life cycle stage of *T. brucei*.

Members of the Kinesin-13 family have been implicated in flagellar length regulation in some organisms. In *Chlamydomonas* CrKinesin-13 is involved in flagellar assembly and disassembly dynamics [8]. After pH-induced flagellar detachment, CrKinesin-13 RNAi-depleted cells showed a delayed phenotype in regenerating a new flagellum, but eventually developed a normal flagellum, congruent with our data indicating that TbKif13-2 is important during the initial stages of flagellar assembly. Activation of flagella assembly in *Chlamydomonas* is concomitant with phosphorylation of CrKinesin-13. In this context it is of interest to note that, in *Leishmania mexicana*, a number of kinases are involved in flagellar length regulation, but their downstream substrates have not yet been identified [18]. Also, in *Giardia lamblia,* a multifunctional Kinesin-13 has been identified that is involved in interphase and mitotic microtubule dynamics, but also in flagellar length regulation [9]. Expression of a kinesin rigor-mutant, presumably incapable of microtubule-depolymerisation due to the inability to hydrolyse ATP, led to a significant elongation of the flagella.

In conclusion, there is now ample evidence that depolymerising kinesins are, in addition to their role in mitosis, also involved in flagellar dynamics. However, the subtle phenotype that we describe for TbKif13-2 is also supportive of a large body of data indicating that flagellar length regulation depends not on a single factor, but is regulated at multiple layers, integrating intraflagellar transport, signalling cascades and microtubule dynamics [19,20].

## Figures and Tables

**Fig. 1:**
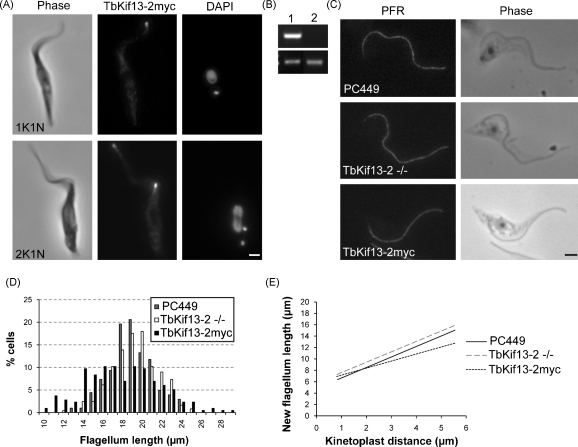
TbKif13-2 localisation and the effect of TbKif13-2 knockout in procyclic *T. brucei* cells. (A) Immunofluorescence of TbKif13-2myc induced cells, fixed with 3.7% (w/v) formaldehyde in PBS. Fixed cells were permeabilised with 0.1% (v/v) NP40 and labelled with anti-myc antibody and stained with DAPI. Cells with a single (1K1N) and a mature and new (2K1N) flagellum are shown. Bars, 2 μm. (B) RT-PCR from cDNA made from total RNA isolated from (1) Control PC449 cells (2) TbKif13-2 knockout cells. The upper panel shows the absence of TbKif13-2 transcript in knockout cells and the lower panel represents a control PCR reaction using the cohesin subunit SMC3 transcript. (C) Trypanosome cytoskeletons labelled with L8C4 which stains the PFR. Cytoskeletons were prepared by extracting whole trypanosome cells with 0.1% (v/v) NP40 before fixation with 3.7% (w/v) formaldehyde in PBS. Bar, 2 μm. (D) Histogram of flagellar length distribution in WT PC449 cells, TBKif13-2 knockout (−/−) cells and TbKif13-2myc induced cells. Only mature flagella in 1N1K cells were scored. (E) Lines of best fits illustrating the increase in the growing new flagellum in relation to the distance between dividing kinetoplasts. Only cells where the two kinetoplasts can be easily be resolved were measured. The plot reveals that there is no change in the rate of flagellar outgrowth between PC449 cells and TbKif13-2 knockout cells but TbKif13-2myc expression results in a decrease in the rate of flagellum outgrowth. Additional information is in the Supplementary data files.
