# Supplementary material for: The role of the Kinesin-13 family protein TbKif13-2 in flagellar length control of Trypanosoma brucei
Source: Mol Biochem Parasitol. 2010 Dec;174(2):137–40. doi: 10.1016/j.molbiopara.2010.08.001 (PMC2984613; doi:10.1016/j.molbiopara.2010.08.001)
Supplement: Supplementary file 1 [file mmc1.pdf]

Supplementary data

Materials and Methods

Trypanosome cell lines and *in vitro* culture

Procyclic strain 29-13 and 449 was maintained in a semi-defined medium (SDM-79), at 28 °C (Brun and Schonenberger, 1979). 449 has the pHD449 plasmid integrated into the ribosomal spacer locus and expresses the Tet repressor (*TetR*) gene which was maintained with 0.5 μg ml^{-1} of phleomycin (Biebinger et al., 1997). The 29-13 cell line, expressing T7 RNA polymerase and Tet repressor, was grown in the presence of 50 μg ml^{-1} of hygromycin and 15 μg ml^{-1} of G418 (Wirtz et al., 1999). Transfection of procyclic strains were done as described in (Beverley and Clayton, 1993).

Generation of TbKif13-1 RNAi cell line

The RNAi construct, $p2T7^{177}$-Tbkif13-2 was made using the $p2T7^{177}$ vector which allows for the tetracycline inducible production of dsRNA. This vector integrates to the transcriptionally silent 177 bp repeat region of the minichromosomal population in the trypanosome genome (Wickstead et al., 2002). A 434 bp DNA fragment (nucleic acid residue 1,328 – 1,761) of the TbKif13-2 open reading frame was PCR-amplified using the sense primer 5'-GGATCCATCTCTAGTGCGCTGTCCGT-3'and anti-sense primer 5'-AAGCTTCAAGTGGCCCTAAGGGTTTT-3'. The PCR fragment was cloned into the RNAi vector using the restriction sites HindIII and BamHI (underlined). The resulting construct was linearised with NotI and transfected into procyclic 29-13 cells by electroporation. Transformants were selected with phleomycine (5 μg ml^{-1}). RNAi was induced by the addition of 1 μg ml^{-1} of doxycycline.

Generation of TbKif13-2myc cell line

Constructs of the C-terminally 2xmyc-tagged TbKif13-2myc kinesin were generated using the tetracycline inducible expression plasmid pHD1484 (Colasante et al., 2006). The entire open reading frames of TbKif13-2 were PCR-amplified using primers ACGGGCCCAGGGCATGACCTCACTCTGTCC and CGGGATCCCACGCTTTCAAGTTCATGAAGCTTTG. The PCR fragments were cloned into the pHD1484 using the restriction sites Apa1/BamH1 (underlined). The NotI-linearised expression construct was electroporated into the procyclic strain 449 cell line, expressing Tet-repressor. The selection of stable transfectants, integrated into the ribosomal RNA gene locus was done with hygromycin at 50 μg ml^{-1}. Expression of the tagged kinesin was induced by the addition of 1 μg ml^{-1} of doxycycline. Cells were analysed 24 hours after induction.

Immunofluorescence microscopy

T. brucei cells were fixed in suspension with 3.6 % formaldehyde in PBS and processed as described (Bessat and Ersfeld, 2009). In addition to the rabbit antiTbKif13-2 antibody, mouse monoclonal L8C4 antibody was used to stain the PFR (Kohl et al., 1999) and the mouse mAb anti-cmyc (clone 9H10, 1:100 dilution, Serotec) to stain myc-tagged kinesin. Cells were examined on an Olympus IX71 epifluorescence microscope equipped with a CCD-camera (F-View, Olympus). Images were pseudo-coloured and assembled in Adobe Photoshop CS4.

Generation of the double knockout TbKif13-2 -/- cell line

The wild-type PC449 cell line was used for the deletion of both endogenous copies of TbKif13-2. Both TbKif13-2 alleles were replaced via homologous recombination using two different antibiotic resistance cassettes conferring resistance to either blasticidin or G418. Selection of clones was with G418 (15 µg ml^{-1}), and blasticidin (20 µg ml^{-1}). The TbKif13-2 5' UTR (nucleic acid residue 399 upstream of the TbKif13-2 start codon to residue 45 into the TbKif13-2 ORF) and 3' UTR (nucleic acid residue 14 to residue 551 downstream of the TbKif13-2 stop codon) was used in the construction of both BSD and NEO resistance cassettes were amplified via standard PCR on genomic DNA from strain 427 using the primers specified in Table S3. These PCR fragments were cloned into the regions flanking either the BSD or NEO gene resistance marker using the restriction sites ApaI, BamHI, SacI and SpeI (Colasante et al., 2006). Deletion of the TbKif13-2 gene was confirmed via Southern blotting and RT-PCR.

RT-PCR

The RNA extracts of the procyclic form of *T. brucei* cells were obtained using the RNeasy Mini Kit (QIAGEN). The RNA extract was further cleaned using an on-column DNase digestion kit (QIAgen). The resulting purified RNA extract was used in the production of cDNA using QIAGEN Omniscript RT Kit. The production of cDNA was done as suggested by the manufacturer using anchored oligo dT-primers from (Thermo Scientific). The resulting cDNA was used as substrate in a standard PCR reaction using appropriate primers.

Flagellar measurements

Live trypanosome cells freshly harvested were resuspended in PBS and allowed to attach to poly-L-lysine coated slides for 5 minutes. The attached cells were treated with ice cold PBS containing 0.1% NP40 for 30 seconds before being fixed with 3.6% formaldehyde in PBS at room temperature for 15 minutes. The cells were then labelled with an mouse anti-paraflagellar rod (PFR) antibody, L8C4 (Kohl et al., 1999) and processed as described in the immunofluorescence microscopy section. A Student's t-test was used to examine of the average flagellar length of the control PC449 cells were significantly different from the TbKif13-2 knockout and TbKif13-2myc overexpressors because the distribution of the flagella lengths obtained were observed to have a normal distribution. An ANCOVA test was performed to examine of the line of best fits of control, TbKif13-2myc induced and TbKif13-2 knockout cells were significantly different because the flagella measurements are assumed to have a normal distribution and that the covariate (kinetoplast distance) is independent factor that directly determines the length of the flagellum. Statistical analysis was performed with a statistical analysis software package SPSS V17.

References

Bessat, M., and K. Ersfeld. 2009. Functional characterisation of cohesin SMC3 and separase and their roles in the segregation of large and minichromosomes in Trypanosoma brucei. *Mol Microbiol*.

Beverley, S.M., and C.E. Clayton. 1993. Transfection of Leishmania and Trypanosoma brucei by electroporation. *Methods Mol Biol*. 21:333-48.

Biebinger, S., L.E. Wirtz, P. Lorenz, and C. Clayton. 1997. Vectors for inducible expression of toxic gene products in bloodstream and procyclic Trypanosoma brucei. *Mol Biochem Parasitol*. 85:99-112.

Brun, R., and Schonenberger. 1979. Cultivation and in vitro cloning or procyclic culture forms of Trypanosoma brucei in a semi-defined medium. Short communication. *Acta Trop*. 36:289-92.

Colasante, C., V.P. Alibu, S. Kirchberger, J. Tjaden, C. Clayton, and F. Voncken. 2006. Characterization and developmentally regulated localization of the mitochondrial carrier protein homologue MCP6 from Trypanosoma brucei. *Eukaryot Cell*. 5:1194-205.

Kohl, L., T. Sherwin, and K. Gull. 1999. Assembly of the paraflagellar rod and the flagellum attachment zone complex during the Trypanosoma brucei cell cycle. *J Eukaryot Microbiol*. 46:105-9.

Wickstead, B., K. Ersfeld, and K. Gull. 2002. Targeting of a tetracycline-inducible expression system to the transcriptionally silent minichromosomes of Trypanosoma brucei. *Mol Biochem Parasitol*. 125:211-6.

Wirtz, E., S. Leal, C. Ochatt, and G.A. Cross. 1999. A tightly regulated inducible expression system for conditional gene knock-outs and dominant-negative genetics in Trypanosoma brucei. *Mol Biochem Parasitol*. 99:89-101.

Supplementary Tables S1-S3

Table S1: Descriptive statistics of the mean, standard deviation and number of flagellum measurements taken for each of the three different cell types. The Student t-test was performed to compare the mean flagellum length of WT cells against the different treatments. The values denoted by a (*) shows that there was a statistically significant difference in average flagellum length.

Cell line	Mean Flagellar Length ± SD(µm)	N	p Value
PC449 (WT)	19.4 ± 2.2	204	1
TbKif13-2 -/-	19.8 ± 2.3	245	0.065
TbKif13-2myc	18.5 ± 3.8	216	0.002*

Table S2: Descriptive statistics on the mean and standard deviation of the slope and y-intercept of the regression lines drawn in Figure 1E and Figure S1B. The Student t-test was performed to compare the slope of the regression lines of WT cells against the different treatments. A post hoc test with a Sidak correction was performed to determine if the regression lines of WT cells against the different treatments were significantly different. The values denoted by a (*) shows that there was a statistically significant difference.

	Slope ± S.E.	Slope p value against PC449 (t-test)	Constant ± S.E. (µm)	N	Regression p value against PC449 (Sidak)
PC449	1.84 ± 0.14	1	4.81 ± 0.45	102	1
TbKif13-2 -/-	1.82 ± 0.22	0.938	5.77 ± 0.62	83	0.001*
TbKif13-2myc	1.24 ± 0.16	0.005*	5.91 ± 0.50	83	0.017*

Table S3: The forward and reverse primer sequences used in the construction of the *BSD* and *NEO* resistance cassettes. The sequences underlined represents the restriction sites ApaI, BamHI, SacI and SpeI that were used for the generation of the knock out cassettes.

UTR region	Primer type	Sequence (5' to 3')
5'	Forward	<u>GAGCTC</u>TGATGTTTGTTTGCTCGTTTGTGCC
	Reverse	<u>ACTAGT</u>ACAGCCACCAGCTGTCGTACC
3'	Forward	<u>GGATCC</u>ATCACATGCCTCAGTGCGCAC
	Reverse	<u>GGGCCC</u>ATGCACAGTTGCATACTCGTAACAC

<u>Legend to Supplementary Figure S1</u>

Figure S1: (A) Southern blot analysis of genomic DNA of wild-type PC449, single knockout TbKif13-2 +/- and double knockout TbKif13-2 -/- cell lines. The genomic DNA was digested with XhoI / HindIII and probed using the 5'UTR of TbKif2. The deletion of the native TbKif2 gene resulted in the shift from ~1100 kb to ~700 kb. (B) A scatter plot illustrating the different data points used in the generation of the lines of best fit (Fig. 1) used to calculate the rate of new flagellum outgrowth in relation to the distance between the two kinetoplasts in dividing cells.

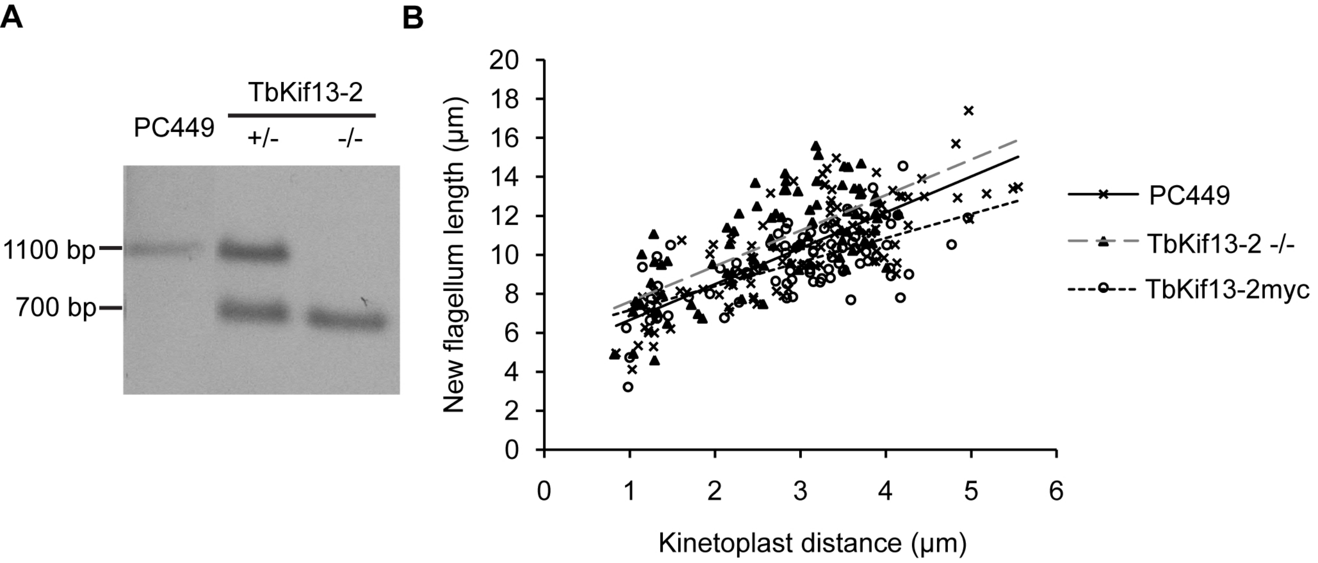
A
PC449
TbKif13-2
+/-
-/-
1100 bp
700 bp
B
New flagellum length (μm)
Kinetoplast distance (μm)
PC449
TbKif13-2 -/-
TbKif13-2myc
0
1
2
3
4
5
6
0
2
4
6
8
10
12
14
16
18
20
